# Aging during *C. elegans* L1 quiescence

**DOI:** 10.18632/aging.104027

**Published:** 2020-09-27

**Authors:** Alejandro Mata-Cabana, Francisco Javier Romero-Expósito, María Olmedo

**Affiliations:** 1 Departamento de Genética, Facultad de Biología, Universidad de Sevilla, Sevilla 41012, Spain

**Keywords:** L1 aging, quiescence, *C. elegans*, insulin signalling

When *C. elegans* embryos hatch in the absence of food, development arrests at the first larval stage (L1). This process involves quiescence of the cells that normally divide at the beginning of postembryonic development. When arrested L1s encounter food, cell divisions resume and postembryonic development proceeds. For this reason, this stage of arrest is routinely used in many *C. elegans* labs to synchronize larva. L1 arrest was described as a non-aging state, since the time spent on it did not account towards the total lifespan once the animals resumed development [1]. However, Roux et al. showed that L1 larvae undergo a process similar to adult aging. Remarkably, the signals of aging accumulated during arrest were erased upon feeding of the arrested larvae [2], explaining why the time spent in arrest does not reduce the lifespan of the adult. Clearance of aging signs seemed necessary to resume development. Although it was somehow implicit that the extent of aging sings would mark the time necessary to recover, the lack of quantitative methods to assess the timing of recovery prevented testing this idea.

Now, we have benefited from a quantitative method that we developed to measure larval developmental timing [3] to quantify the time to recover after prolonged L1 arrest. In a recent publication [4], we have shown that markers of aging that accumulate during L1 arrest contribute to increase the time needed to recover after prolonged arrest. Furthermore, increased activity of the transcription factor DAF-16, as that found in the insulin receptor mutant daf-2, led to faster recovery. Conversely, daf-16 mutants showed strong delays in recovery, even after short periods of arrest. This role of Insulin signaling was surprising, as activation of DAF-16 is commonly related to repression of proliferation. daf-16 mutants are defective in L1 arrest and a fraction of the larvae in the population initiate postembryonic cell divisions, what was attributed to a lower activation of the cyclin dependent kinase inhibitor cki-1 [5]. However, this only happens when there is ethanol present in the media of arrested L1, normally used to dilute the cholesterol supplementation for C. elegans. Instead, we used media without ethanol and observed the same levels of cki-1 expression in the wild-type strain and daf-16 mutants. Analysis of the initial divisions of postembryonic development revealed that the daf-16 mutant not only did not show premature divisions, but they suffered an important delay compared to the wild type. Then, how do we explain the repression of cell divisions in the daf-16 mutant? Our hypothesis was that insulin signaling modulates the rate of aging during L1 arrest, as it does for adult aging [6]. Indeed, we could confirm that activation of DAF-16 reduces the rate of L1 aging. Therefore, the delay in the initiation of postembryonic development in the daf-16 mutant it is not related to a direct role of this transcription factor in the control of cell divisions during recovery but to the lack of activation of the stress response during L1 arrest, leading to a faster rate of L1 aging. The faster accumulation of L1 aging markers entails longer recovery times to clear them, delaying the initiation of postembryonic divisions [4].

**Figure 1:**
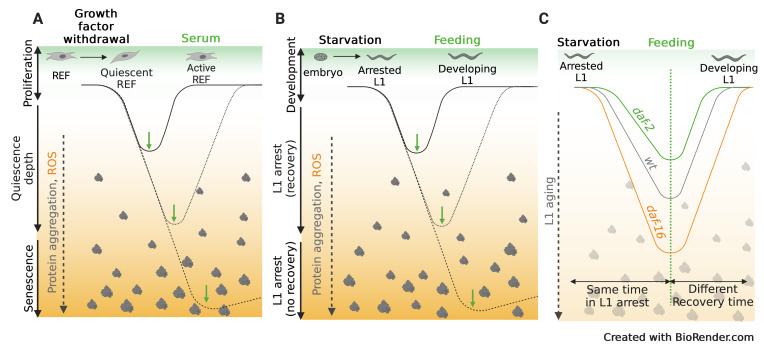
**Parallelisms between quiescence deepening and L1 ageing. (A)** Upon removal of growth factors, REF (Rat Embryonic Fibroblast) enter cell quiescence, a reversible, non-proliferative state. Cells progressively undergo quiescence deepening as they accumulate ROS and protein aggregates. After prolonged quiescence, cells require stronger stimulation and take longer to exit quiescence and return to proliferation. Eventually cells reach senescence, an irreversible state of cell arrest. **(B)** L1 arrested *C. elegans* larvae show a similar process to quiescence deepening. Prolonged arrest entails a process named L1 aging, that also involves accumulation of ROS and protein aggregates. We have shown that prolonged quiescence leads to a delay in the initiation of postembryonic cell divisions. Before dying as arrested L1, larvae lose the capacity to recover from L1 arrest, a process that could be compared to cell senescence. **(C)** Insulin signalling modulated the process of L1 aging. Activation of the transcription factor DAF-16, as that sound in *daf-2* mutants, leads to a slower rate of L1 aging. As a consequence, after the same time in L1 arrest, *daf-2* mutants accumulate less markers of aging. This allows a faster recovery once larvae encounter food. *daf-16* mutants, show faster L1 ageing and the initiation of postembryonic divisions upon feeding is delayed compared to the wild type.

The lack of homogeneity of L1 arrest resembles the process of quiescence deepening undergone by mammalian cells. Capacity of quiescent cells to recover proliferation potential depends on the depth of quiescence. This process has been studied in detail in Rat Embryonic Fibroblasts. Cells in shallow quiescence respond faster to growth stimuli than those in deep quiescence, which need stronger stimuli and longer times to initiate proliferation. Deep quiescent cells share gene-expression signatures with senescent and aged cells, what suggests quiescence deepening as a transitional state toward senescence and aging. Cells cultured for long periods under quiescence conditions move from shallow to deep quiescence and, ultimately, they irreversibly arrest as senescent cells [7]. Similar to what we have described in *C. elegans* for arrested L1s [4], accumulation of signs of aging during prolonged periods of quiescence delays the reactivation of cell proliferation. Lysosomal function controls quiescence deepening by reducing the accumulation of Reactive oxygen species (ROS) [7]. Cells subjected to long-term serum starvation underwent lysosomal function impairment leading to a higher ROS accumulation, resulting in a loss of capacity to reenter cell cycle [7]. In this way, when quiescent cells were pharmacologically treated to inhibit lysosomal function or to induce oxidative stress, they were pushed into a deeper quiescent state and they needed stronger stimulation to recover [7]. As ROS, accumulation of protein aggregates also leads to quiescence deepening. Lysosomal activation is also important to clear protein aggregates in quiescent cells to keep them in a shallower quiescent state [8]. This is in concordance with our findings, where arrested larvae with higher levels of ROS and protein aggregation needed longer times to recover and to initiate post-embryonic development. This leads us to raise the idea of the quiescence depth in the context of *C. elegans* and to propose arrested L1 larvae as a model for the study of this process. The control of the quiescence depth is fundamental to maintain the tissue regenerative potential, but also, the dysregulation of the cellular quiescence can result in hyperproliferation that leads to cancer (Figure 1). Therefore, the understanding of the molecular mechanisms underlying this phenomenon is of special interest and the *C. elegans* model is a useful tool to gain knowledge about these processes.
